# LncRNA CCAT1 facilitates the proliferation, invasion and migration of human laryngeal squamous cell carcinoma cells *via* the miR-218-5p/BMI1

**DOI:** 10.7717/peerj.12961

**Published:** 2022-03-03

**Authors:** Jing Hong, Ali Hong, Houshu Tu, Zhichao Wan, Yuqiao Deng, Chengcheng Deng, Bo Tao, Yanjin Yu, Lanfei Zhou

**Affiliations:** 1 Affiliated Hospital of Jiangxi University of Traditional Chinese Medicine, Nanchang, China; 2 Jiangxi University of Traditional Chinese Medicine, Nanchang, China; 3 Nanchang Angel Maternity Hospital, Nanchang, China

**Keywords:** Laryngeal squamous cell carcinoma, LncRNA CCAT1, miR-218-5p, BMI1, Competing endogenous RNA, Proliferation, Migration, Invasion

## Abstract

Long non-coding RNAs (LncRNAs) are vital in the treatment of laryngeal squamous cell carcinoma (LSCC). This study estimated the mechanism of lncRNA CCAT1 (CCAT1) in LSCC cells. The expression of CCAT1 in the human laryngeal mucosal epithelial cells (HLCs) and LSCC cells (Hep-2 and TU177) was detected. CCK-8 and Transwell assays were used to evaluate the cell proliferative, migrative, and invasive abilities, respectively. The subcellular localization of CCAT1 was verified by RNA-FISH and cytoplasmic isolation assays. The targeted relationship among CCAT1, miR-218-5p, and BMI1 was verified by dual-luciferase assay. Expressions of miR-218-5p and BMI1 were detected by RT-qPCR. Our results depicted that CCAT1 was highly-expressed in Hep-2 and TU177 cells. Silencing CCAT1 inhibited the proliferation, migration, and invasion of Hep-2 and TU177 cells. Mechanically, CCAT1 regulated the BMI1 expression by competitively binding to miR-218-5p as a competing endogenous RNA (ceRNA), and thus facilitated the growth of Hep-2 and TU177 cells. Downregulation of miR-218-5p or upregulation of BMI1 inhibited the inhibitory effect of silencing CCAT1 on Hep-2 and TU177 cell proliferation, invasion, and migration. In conclusion, our study elicited that lncRNA CCAT1 facilitated the proliferation, migration, and invasion of Hep-2 and TU177 cells by sponging miR-218-5p and regulating the downstream BMI1.

## Introduction

Laryngeal squamous cell carcinoma (LSCC) is a subtype of head and neck squamous cell carcinoma with an alarming incidence (Mastronikolis et al., 2020). LSCC has the second-highest mortality rate amongst all respiratory malignant tumors (Siegel, Miller & Jemal, 2016). Common manifestations of LSCC comprise voice alterations, difficulty in breathing and swallowing, and obstruction of the airway vocalization, which further deteriorates the quality of life (Johnson et al., 2020). Some associated risk factors of LSCC are chronic alcohol consumption, tobacco, and infection with Human Papilloma Virus (HPV) (Tsiambas et al., 2020). The currently available treatment modalities for LSCC consist of radiotherapy, surgery, and chemotherapy (Kitamura et al., 2020). However, we still incur significant challenges in treating LSCC due to its underlying complications of frequent recurrence and metastasis (von Witzleben et al., 2020). Therefore, significant efforts are warranted for insight into the treatment of LSCC.

Long non-coding RNAs (LncRNAs) are regulatory RNAs that range over 200 nt in length and could interact with DNA, RNA, or protein molecules (Peng, Koirala & Mo, 2017). LncRNAs can radically manipulate several cellular processes in different diseases and cancers (Zhang & Tang, 2018). Recent investigations have elicited the regulatory effects of several lncRNAs including IUR, WTAPP1, and FER1L4 on cell migration, proliferation, and invasion in LSCC (Jiao et al., 2020; Shi et al., 2020; Wei et al., 2020). Among the numerous lncRNAs, lncRNA colon cancer-associated transcript-1 (CCAT1) has attracted our attention. CCAT1 is classified as an oncogenic lncRNA in several human cancers (Guo & Hua, 2017). An abnormal expression of CCAT1 is often associated with the progression and metastasis of tumors and patient survival, with the involvement of different target genes and signaling pathways in the regulatory metabolic processes (Liu, Chen & Hann, 2019). Intriguingly, CCAT1 can expedite cell proliferation and invasion in LSCC (Zhuang et al., 2016). Moreover, the function of the lncRNA-mediated competing endogenous RNA (ceRNA) network [lncRNA-microRNA (miRNA)-mRNA] is fundamental in the tumorigenesis of LSCC (Liu & Ye, 2019; Lyu et al., 2020). Essentially, CCAT1 could extensively stimulate the development of LSCC by serving as a ceRNA to enhance ZFX, the target of miR-218, by direct inhibition of miR-218 (Zhang & Hu, 2017). A recent study implicated the application of the downregulation of miR-218-5p to facilitate the invasion of oral squamous cell carcinoma cells, implying a potential anti-cancer function of miR-218-5p (Li et al., 2018b). Moreover, miR-218 suppresses the expression of B-cell-specific moloney murine leukemia virus insertion site 1 (BMI1) to subsequently impede cell proliferation and metastasis in esophageal squamous cell carcinoma (Wang et al., 2015). Simultaneously, an elevated BMI1 expression can potentially facilitate tumor formation in oral squamous cell carcinoma and becomes an inducible factor of tumors (Kalish et al., 2020).

In light of the aforementioned findings, we hypothesized that lncRNA CCAT1 mediates cell proliferation and invasion in LSCC by competitively binding to miR-218-5p as a ceRNA to regulate the BMI1 expression. To validate the hypothesis, the current study sought to investigate the *in vitro* functional mechanism of the CCAT1-mediated ceRNA network in LSCC.

## Materials and Methods

### Cell culture

Human laryngeal mucosal epithelial cells (HLCs) were provided by iCell Bioscience Inc. (Shanghai, China). Human LSCC cells Hep-2 and TU177 were provided by ATCC (Manassas, VA, USA) and Bena Culture Collection Co., Ltd. (Beijing, China), respectively. Briefly, the cells were meticulously cultured in DMEM (Thermo Fisher Scientific, San Jose, CA, USA) supplemented with 10% fetal bovine serum (FBS; Gibco, Gaithersburg, MD, USA) and 1% penicillin/streptomycin (Gibco) at 5% CO_2_ and 37 °C.

### Cell transfection

Cells were classified as follows: the HLC group (HLCs), the Hep-2/TU177 group (human LSCC cells), the sh-NC group (Hep-2/TU177 cells transfected with sh-NC), the sh-CCAT1 group (Hep-2/TU177 cells transfected with sh-CCAT1), the mimic-NC group (Hep-2/TU177 cells transfected with mimic-NC), the miR-218-5p mimic group (Hep-2/TU177 cells transfected with miR-218-5p mimic), the inhibitor NC group (Hep-2/TU177 cells transfected with inhibitor NC), the miR-218-5p inhibitor group (Hep-2/TU177 cells transfected with miR-218-5p inhibitor), the sh-CCAT1 + inhibitor-NC group (Hep-2/TU177 cells transfected with sh-CCAT1 and inhibitor-NC), the sh-CCAT1 + miR-218-5p inhibitor group (Hep-2/TU177 cells transfected with sh-CCAT1 and miR-218-5p inhibitor), the oe-NC group (Hep-2/TU177 cells transfected with pcDNA3.1 NC), the oe-BMI1 group (Hep-2/TU177 cells transfected with pcDNA3.1 BMI1), the sh-CCAT1 + oe-NC group (Hep-2/TU177 cells transfected with sh-CCAT1 and pcDNA3.1-NC), and the sh-CCAT1 + oe-BMI1 group (Hep-2/TU177 cells transfected with sh-CCAT1 and pcDNA3.1 BMI1). Additionally, the sh-CCAT1, miR-218-5p mimic, miR-218-5p inhibitor, pcDNA3.1 BMI1, and corresponding controls (GenePharma, Shanghai, China) were introduced into the experimental cells upon attaining 80% confluence using Lipofectamine 2000 (Invitrogen, Carlsbad, CA, USA). Subsequent experimentation was conducted after 48-h transfection.

### Reverse transcription-quantitative polymerase chain reaction (RT-qPCR)

The total RNA content was extracted from the cells in each group using the TRIzol kits (Invitrogen, Waltham, MA, USA) in strict accordance with the provided instructions. The extracted RNA content was synthesized into cDNA using the Primescript RT kits (TaKaRa, Dalian, China). Next, the cDNA was amplified and quantified using SYBR Green mix (TaKaRa, Dalian, China) on the Applied Biosystems 7500 instrument. The primer sequences have been presented in Table 1. The relative gene expression was calculated based on the 2^−ΔΔCt^ method (Hao et al., 2019). GAPDH and U6 served as the internal controls of CCAT1, BMI1 and miR-218-5p, respectively.

**Table 1:** Primer sequences.

Gene	Forward 5′-3′	Reverse 5′-3′
*CCAT1*	TCACTGACAACATCGACTTTGAAG	GGAGAAAACGCTTAGCCATACAG
*miR-218-5p*	TTGCGGATG GTTCCGTCAAGCA	ATCCAGTGCAGGGTCC GAGG
*BMI1*	CCACCTGATGTGTGTGCTTTG	TTCAGTAGTGGTCTGGTCTTGT
*GAPDH*	GGACCTGACCTGCCGTCTAG	GTAGCCCAGGATGCCCTTGA
*U6*	CTCGCTTCG GCAGCACA	AACGCTTCACGAATTTGCGT

**Note:**

CCAT1, colon cancer-associated transcript 1; miR, microRNA; BMI1, B cell-specific Moloney murine leukaemia virus integration site 1; GAPDH, glyceraldehyde-3-phosphate dehydrogenase.

### Western blot

The total protein content was extracted by lysis of cells with the RIPA lysis buffer (Solarbio, Beijing, China) and quantified using the provided BCA kits (Beyotime, Shanghai, China). Subsequently, 50 µg of protein content was separated using 10% SDS-PAGE and transferred onto the PVDF membranes (Thermo Fisher Scientific, Waltham, MA, USA). After 1-h of membrane blockade using 5% skim milk, the membranes were incubated overnight at 4 °C with the corresponding primary antibodies BMI1 (ab38195, at a dilution ratio of 1:1,000; Abcam, Cambridge, United Kingdom) and β-actin (ab115777, at a dilution ratio of 1:200; Abcam, Cambridge, United Kingdom). On the following day, the membranes were rinsed thrice with TBST at room temperature and incubated with the HRP-conjugated goat anti-rabbit secondary antibody IgG H&L (at a dilution ratio of 1:5000, ab97051; Abcam) for 1 h. After 3 TBST rinses, the ECL substrate (Thermo Fisher Scientific, Waltham, MA, USA) was added to detect the protein immunoreactivity using FluorChem E (ProteinSimple, Santa Clara, CA, USA). Gray value of the protein bands was analyzed using the Image-Pro Plus 6.0 software (Media Cybernetics, Rockville, MD, USA) with β-actin as the internal reference.

### Cell counting-kit 8 (CCK-8) assay

CCK-8 kit (Beyotime, Jiangsu, China) was utilized to detect the degree of cell proliferation. Cells were seeded in 96-well plates (5,000 cells/well) and cultured for 48 h, after which 10 μL of the CCK-8 solution was added into each well for 1-h incubation. The absorbance value at the excitation wavelength of 450 nm was detected using a microplate reader (Bio-Rad, Hercules, CA, USA) to assess the cell viability.

### Transwell assay

Cell migrative and invasive abilities were assessed using the Transwell invasion chamber (Becton Dickinson, Bedford, MA, USA). Next, the cells cultured in the serum-free medium (2 × 10^5^) were paved in the apical chamber coated with or without 10 μg/mL matrigel (for invasion or migration assay) and the culture medium with 10% FBS was supplemented to the basolateral chamber as a chemotactic agent. After 24 h, the cells present above the membrane were removed with cotton swabs, while the cells that migrated through the membrane were stained with crystal violet and counted under an Olympus microscope.

### RNA fluorescence *in situ* hybridization (FISH) assay

The DNA probe targeting the end-to-head junction of CCAT1 was labeled using fluorescein isothiocyanate (FITC). The sample was fixed in 4% (wt/Vol) paraformaldehyde, permeabilized in 1 × phosphate buffer saline (PBS) with 0.5% (Vol/Vol) Triton X-100 for 10 min, and then rinsed in 1 × PBS with 0.1% (Vol/Vol) Tween-20 for 1 min. The probe was combined with a pre-made hybridization buffer, followed by overnight incubation with the hybridization buffer at 37 °C. After a 15-min rinse with hybridization buffer at 37 °C and three quick rinses, the cells were stained with 4′,6-diamidino-2-phenylindole solution. Finally, the observations were documented under an immunofluorescence microscope (TCS SP5II, Leica, Wetzlar, Germany).

### Nuclear/Cytosol fractionation assay

The cytoplasm and nucleus were isolated from the Hep-2 and TU177 cells and separated using the provided Nuclear and Cytoplasmic Extraction kits (Beyotime). The cells were rinsed with PBS and collected into 200 μL of cytoplasmic protein extractant A/protease inhibitor buffer and allowed to rest on ice for 10–15 min. Subsequently, the cells were supplemented with 10 μL cytoplasmic protein extractant B and centrifuged at 4 °C and 12,000 g for 10 min to sequester the nuclear precipitation away from the cytoplasm. The nucleus was resuspended using 50 μL of the nucleoprotein extract and mixed on ice for 30 min, followed by 10-min of centrifugation at 4 °C and 12,000 g. The supernatant was isolated as nuclear extract and analyzed by RT-qPCR.

### Dual-luciferase assay

The wild-type (WT) or mutant (MUT) CCAT1 or BMI1 (GenePharma, Shanghai, China) sequences containing the miR-218-5p binding sites were inserted into the pGL3-basic vectors (Promega, Madison, WI, USA). The Hep-2 and TU177 cells were supplemented with the miR-218-5p mimic or corresponding mimic NC to detect the luciferase activity after 48 h using the luciferase reporter assay system (Promega, Madison, WI, USA).

### Statistical analysis

The experimental data were analyzed using SPSS24.0 (IBM Corp., Armonk, NY, USA) and plotted using GraphPad Prism8.0.1 (GraphPad Software Inc., San Diego, CA, USA). Data were presented as mean ± standard deviation (SD). Each experiment was conducted 3 times independently. Pairwise comparisons were analyzed using the independent sample *t* test and comparisons among multi-groups were analyzed using one-way analysis of variance (ANOVA), followed by Tukey’s test. The *p* value was two-sided. In all statistical references, a value of *p* < 0.05 was statistically significant.

## Results

### Silencing CCAT1 inhibited proliferation, invasion, and migration of LSCC cells

**Figure 1:**
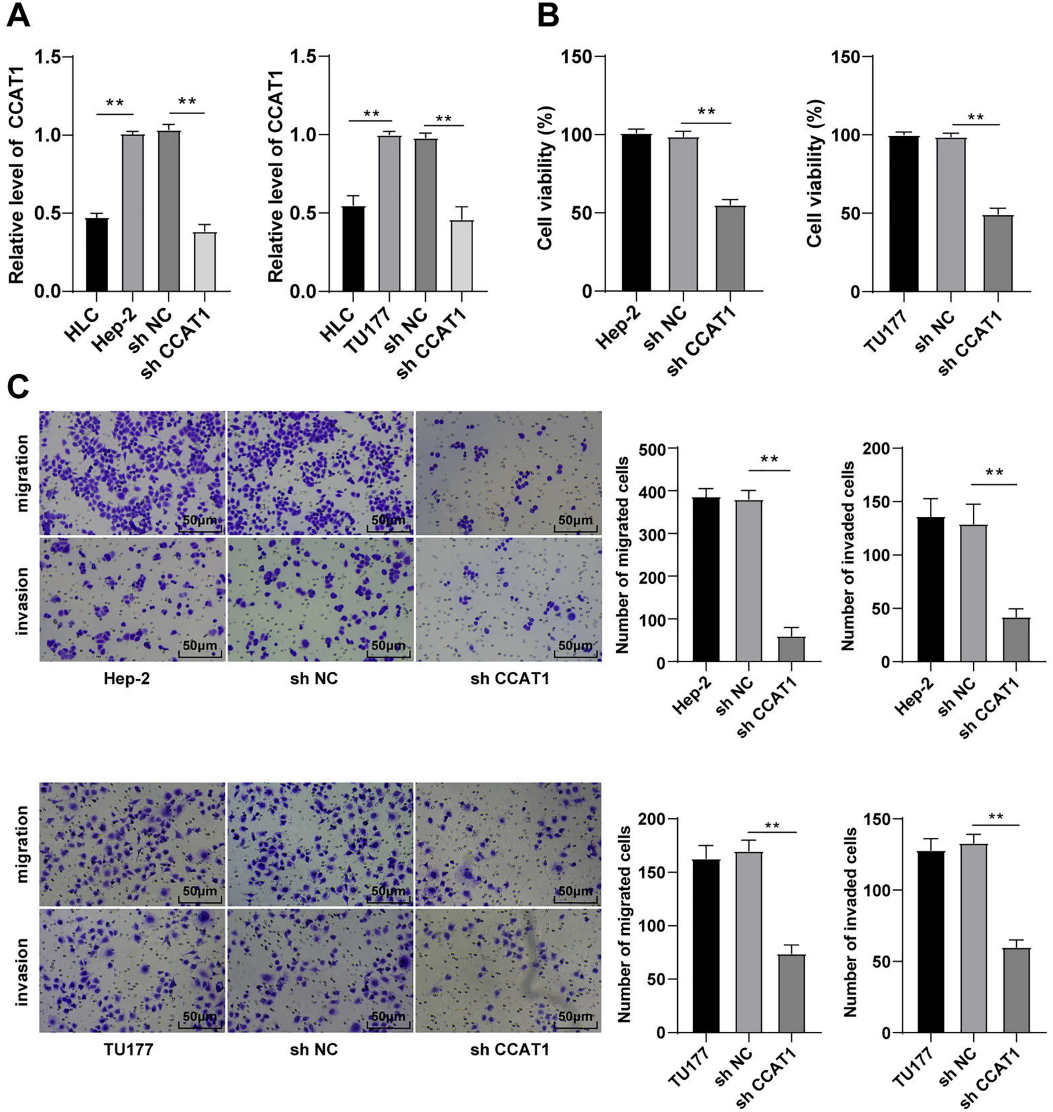
Silencing CCAT1 inhibited proliferation, invasion, and migration of Hep-2/TU177 cells. Note: ** *p* < 0.01.

The involvement of lncRNA CCAT1 is implicated in the development of several cancers with a prominent expression in endometrial cancer, colorectal cancer, and cervical carcinoma (Gu et al., 2020; Treeck et al., 2020; Zhang et al., 2021). Additionally, the upregulation of CCAT1 could significantly improve the survival capacity and metastatic rate of cancer cells. For a comprehensive understanding of the function of CCAT1 in LSCC, we initially compared the expression pattern of CCAT1 in HLCs and human LSCC cells. The results of RT-qPCR exhibited a higher expression pattern of CCAT1 in Hep-2 and TU177 cells relative to the HLCs (*p* < 0.01, Fig. 1A). To further determine the impact of CCAT1 on the proliferation and migration of human LSCC cells, shRNA was delivered into the Hep-2 and TU177 cells to suppress the CCAT1 expression pattern with sh-NC as a control. A reduced expression pattern of CCAT1 has been evident in the Hep-2 and TU177 cells after transfection with sh-CCAT1 (*p* < 0.01, Fig. 1A). The findings of CCK-8 assay showed that silencing CCAT1 inhibited the proliferative ability of Hep-2 and TU177 cells (*p* < 0.01, Fig. 1B). The weakened invasive and migrative abilities of Hep-2 and TU177 cells were detected by Transwell assays after silencing CCAT1 (all *p* < 0.01, Fig. 1C). The aforementioned results demonstrated that silencing CCAT1 inhibited the malignant behaviors of Hep-2 and TU177 cells.

### CCAT1 directly interacted with miR-218-5p in LSCC cells

**Figure 2:**
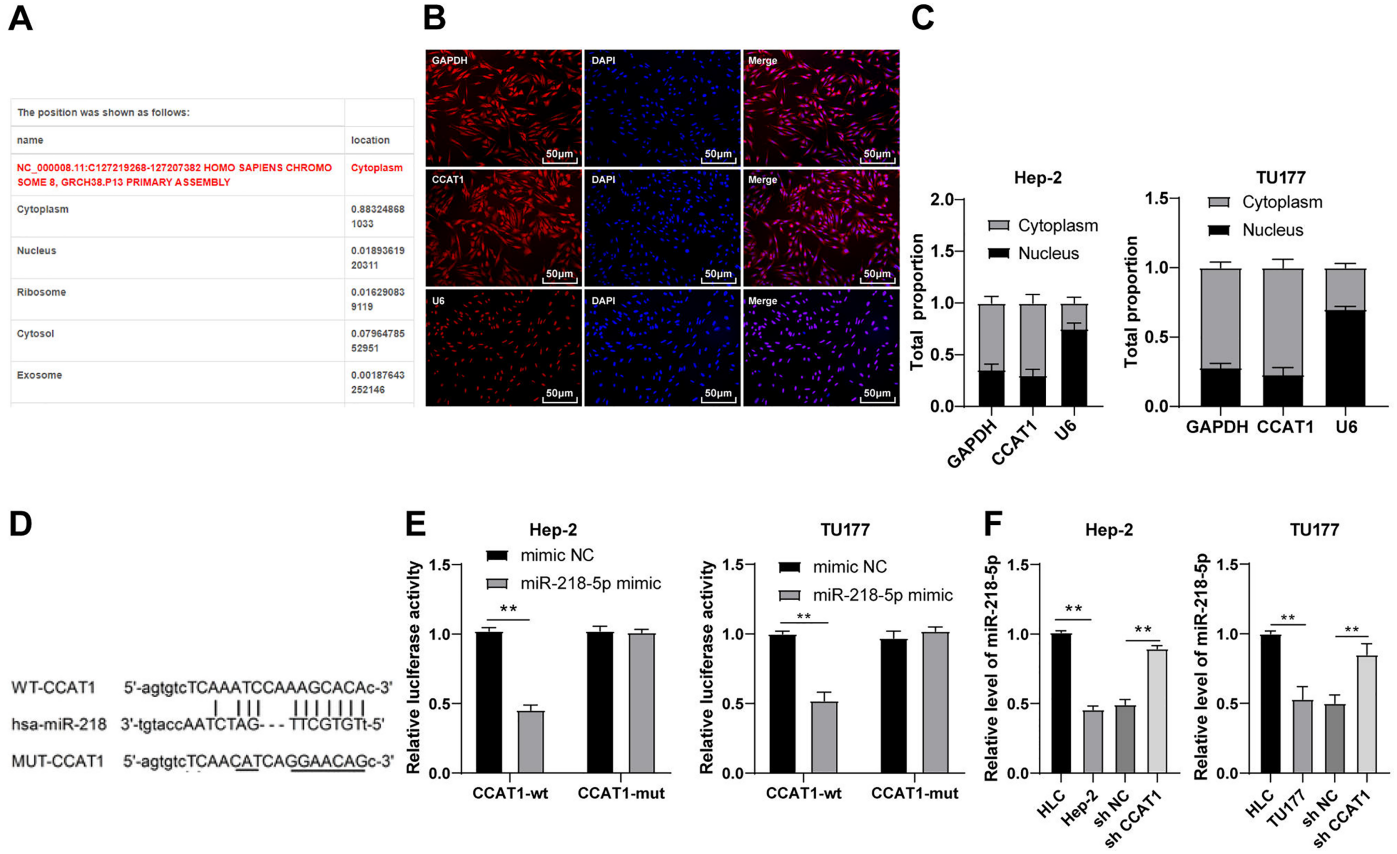
CCAT1 directly interacted with miR-218-5p in Hep-2/TU177 cells. Note: ** *p* < 0.01.

To investigate the regulatory mechanism of CCAT1, we predicted the subcellular localization of CCAT1 on the bioinformatics website (http://www.csbio.sjtu.edu.cn/bioinf/lncLocator/) and identified the predominant localization of CCAT1 in the cytoplasm (Fig. 2A), which was further verified by RNA-FISH and nuclear/cytosol fractionation assay (Figs. 2B and 2C). Therefore, we speculated the ability of CCAT1 to regulate the biological process of human LSCC cells by functioning as a ceRNA. To prove this hypothesis, the binding sites of CCAT1 and miR-218-5p were predicted on the Starbase database (http://starbase.sysu.edu.cn/agoClipRNA.php?source=lncRNA) (Fig. 2D). Previously, the participation of miR-218-5p has been documented in the development of human LSCC (Zhang & Hu, 2017). We subsequently verified the targeted relationship between CCAT1 and miR-218-5p by dual-luciferase assay, which exhibited decreased luciferase activity in cells transfected with miR-218-5p mimic and CCAT1-WT while no significant difference was apparent in cells transfected with miR-218-5p mimic and CCAT1-MUT (*p* < 0.01, Fig. 2E). Furthermore, the results of RT-qPCR exhibited a lower expression pattern of miR-218-5p in the Hep-2 and TU177 cells than that in HLCs, and a higher expression pattern of miR-218-5p in Hep-2 and TU177 cells transfected with sh-CCAT1 than that in cells transfected with sh-NC (all *p* < 0.01, Fig. 2F). The aforementioned findings implicated the application of CCAT1 as a ceRNA of miR-218-5p.

### Downregulation of miR-218-5p partially averted the inhibitory effect of silencing CCAT1 on Hep-2/TU177 cells

**Figure 3:**
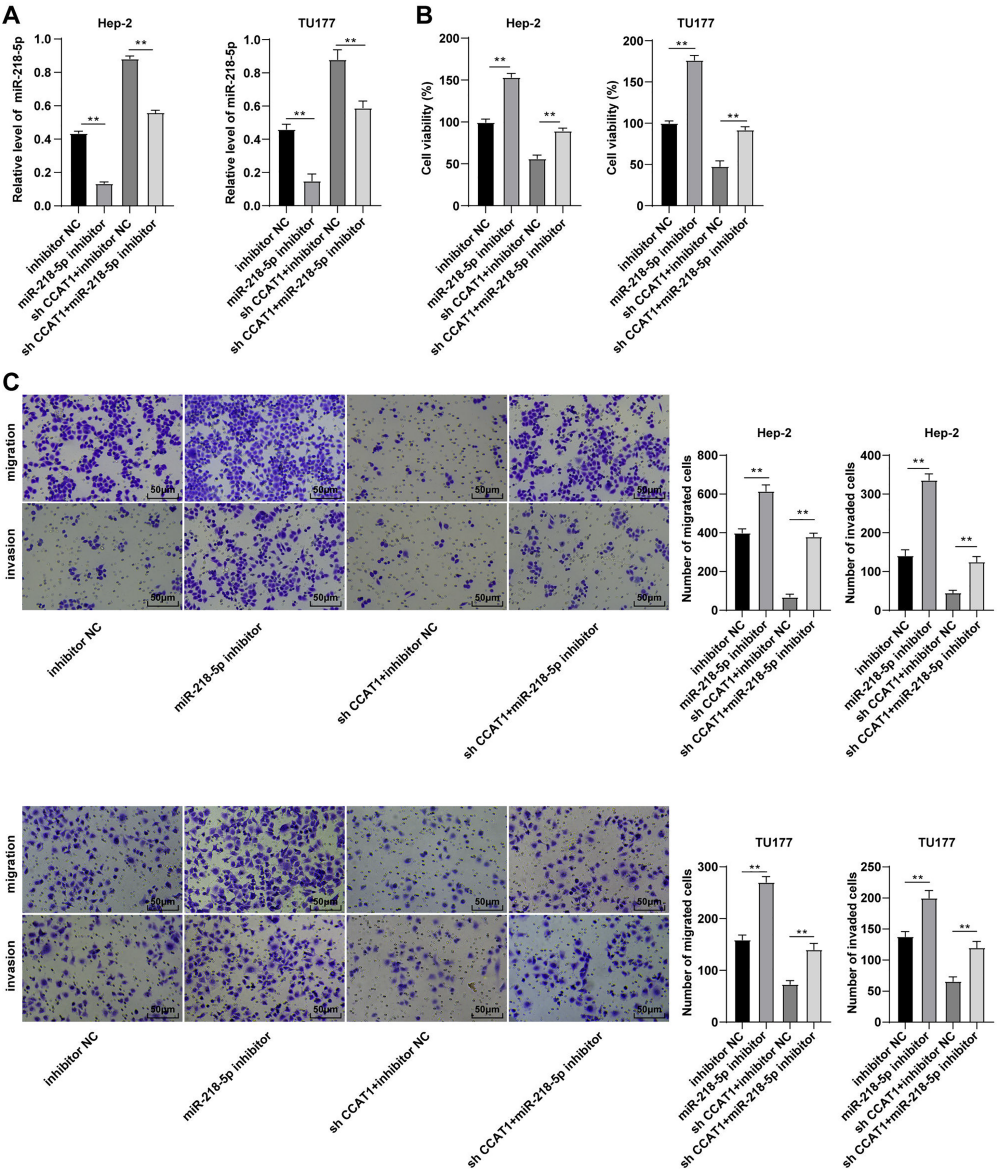
Downregulation of miR-218-5p partially averted the inhibitory effect of silencing CCAT1 on Hep-2/TU177 cells. Note: ** *p* < 0.01.

To validate whether CCAT1 could modulate the Hep-2 and TU177 cells by competitively binding to miR-218-5p, a rescue experiment was conducted to deliver miR-218-5p inhibitor into the Hep-2 and TU177 cells with silencing CCAT1. The results of RT-qPCR revealed that the miR-218-5p expression pattern was at the lowest level in cells transfected with miR-218-5p inhibitor only and was notably increased after silencing CCAT1 (all *p* < 0.01, Fig. 3A). Subsequently, CCK-8 assay determined that the miR-218-5p inhibitor significantly promoted cell proliferation while the downregulation of miR-218-5p could partially annul the alterations in cell proliferation induced by sh-CCAT1 (all *p* < 0.01, Fig. 3B). Similarly, Transwell assays demonstrated that transfection with the miR-218-5p inhibitor solemnly significantly facilitated cell migration and invasion, while the downregulation of miR-218-5p could partially reverse the suppression of silencing CCAT1 on cell invasion and migration (all *p* < 0.01, Fig. 3C). Based on these findings, CCAT1 could promote the biological behaviors of Hep-2 and TU177 cells by competitively binding to miR-218-5p.

### miR-218-5p targeted BMI1

**Figure 4:**
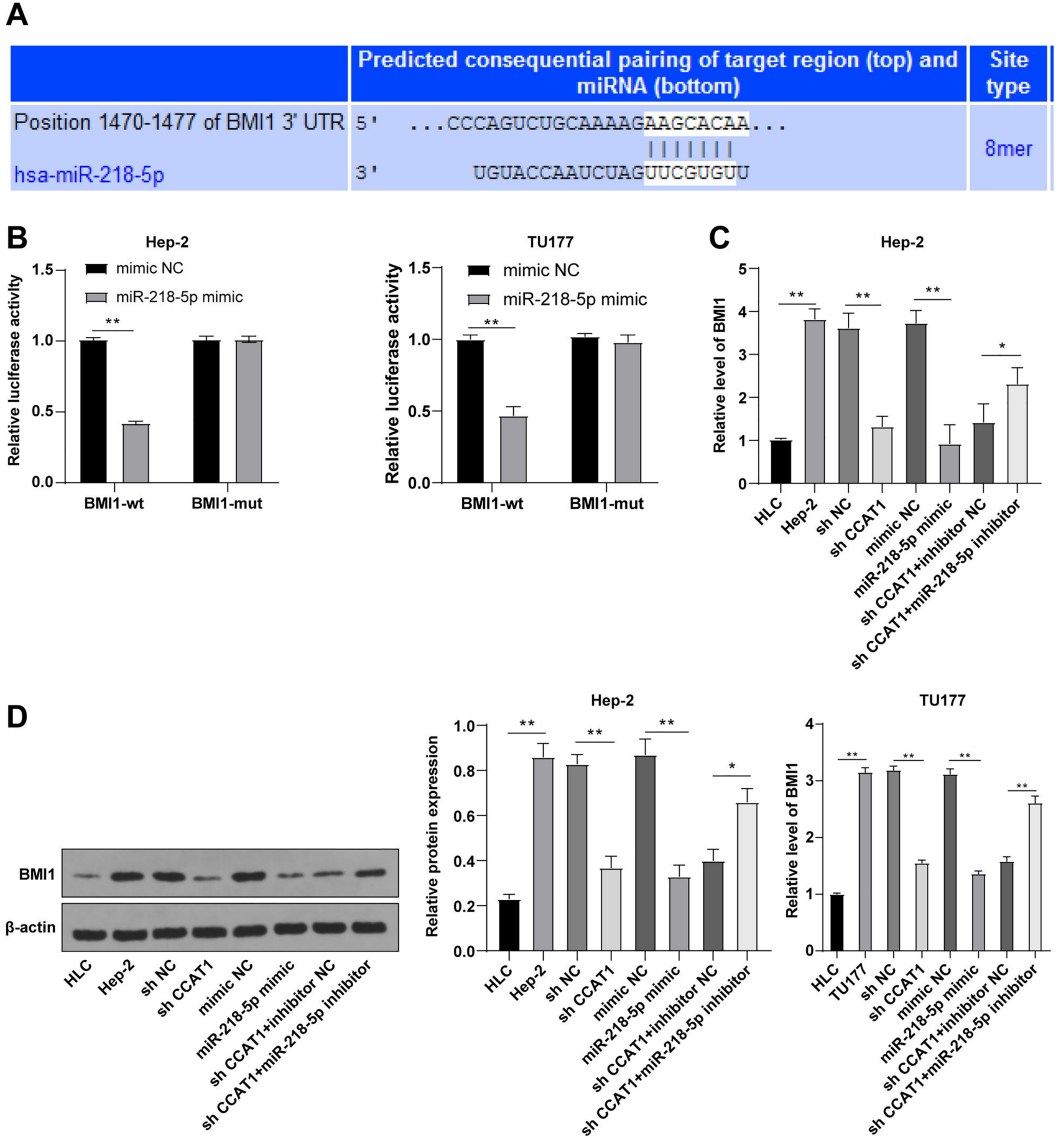
miR-218-5p targeted BMI1. Note: * *p* < 0.05, ** *p* < 0.01.

To further investigated the downstream target gene regulated by CCAT1 as the ceRNA of miR-218-5p, a prediction conducted on the Targetscan database (http://www.targetscan.org/vert_71/) identified the corresponding binding sites of miR-218-5p and BMI1 (Fig. 4A). Moreover, the inhibition of BMI1 is implicated as a potential therapeutic option for various cancers (Chen et al., 2011). Dual-luciferase assay was indicative of reduced luciferase activity after transfection with miR-218-5p mimic and BMI1-WT, whereas no significant difference was evident after transfection with miR-218-5p mimic and BMI1-MUT in Hep-2 and TU177 cells (*p* < 0.01, Fig. 4B). The results of RT-qPCR showed that silencing CCAT1 and miR-218-5p overexpression could both decrease the BMI1 expression pattern, and the downregulation of miR-218-5p partially inverted the inhibitory effect of silencing CCAT1 on the BMI1 expression pattern in Hep-2 and TU177 cells (*p* < 0.05, Fig. 4C). The results of Western blot identified the same trend in Hep-2 cells (Fig. 4D). The aforementioned results indicated that miR-218-5p had targeted BMI1 in Hep-2 and TU177 cells.

### Upregulation of BMI1 partially annulled the inhibitory effect of silencing CCAT1 on Hep-2/TU177 cells

**Figure 5:**
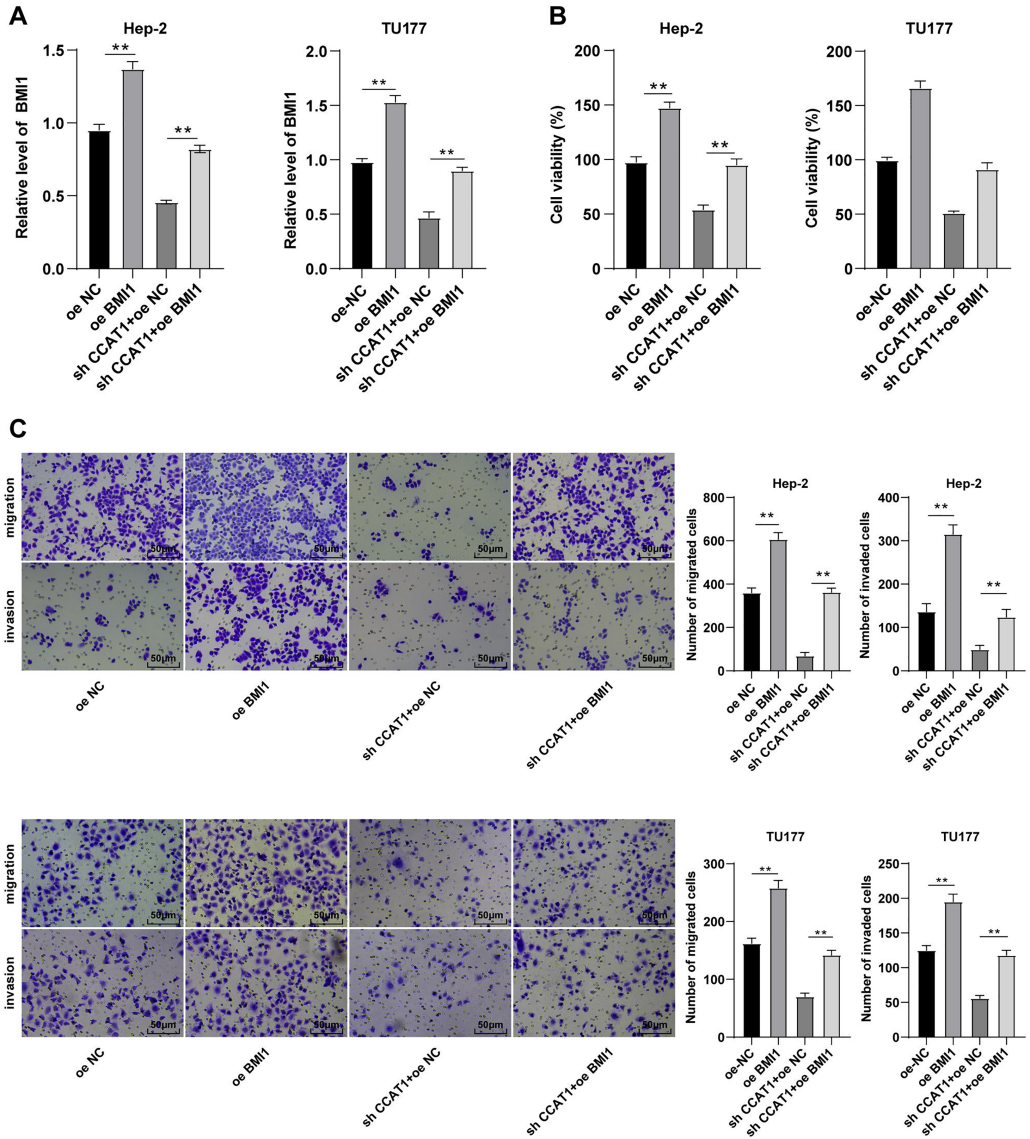
Upregulation of BMI1 partially annulled the inhibitory effect of silencing CCAT1 on proliferation, invasion, and migration of Hep-2/TU177 cells. Note: ** *p* < 0.01.

To further validate the ceRNA mechanism of the CCAT1/miR-218-5p/BMI1 axis in the biological behavior of Hep-2 and TU177 cells, we observed the combined effect of silencing CCAT1 and BMI1 overexpression on the proliferation, invasion, and migration of Hep-2 and TU177 cells through numerous rescue experiments. An elevated expression pattern of BMI1 in Hep-2 and TU177 cells was detected by RT-qPCR after transfection with pcDNA3.1 BMI1 individually, whereas a further BMI1 overexpression partially neutralized the inhibitory function of silencing CCAT1 on BMI1 expression pattern (all *p* < 0.05, Fig. 5A). Subsequently, the CCK-8 assay showed that the upregulation of BMI1 could facilitate Hep-2 and TU177 cell proliferation and reverse the effect of silencing CCAT1 on the proliferation of Hep-2 and TU177 cells (all *p* < 0.05, Fig. 5B). Similarly, the results of Transwell assay exhibited that individual treatment with BMI1 overexpression facilitated Hep-2 and TU177 cell migration and invasion, while the upregulation of BMI1 partially prevented the inhibition of silencing CCAT1 on Hep-2 and TU177 cell invasion and migration (all *p* < 0.01, Fig. 5C). In combination with previous results, our findings concluded that CCAT1 stimulated the growth of Hep-2 and TU177 cells by sponging miR-218-5p to facilitate the BMI1 expression.

## Discussion

**Figure 6:**
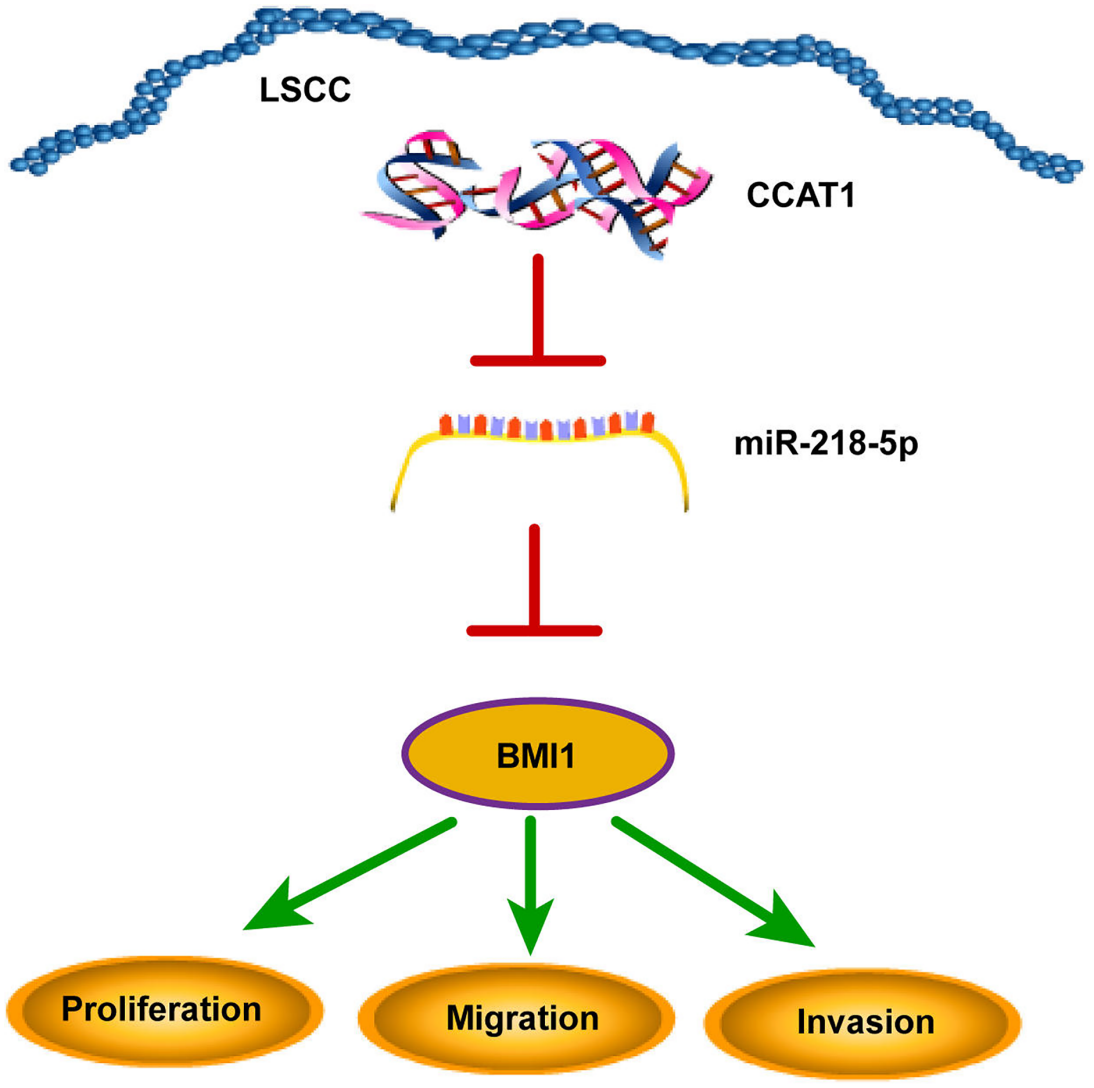
CCAT1 facilitated the proliferation, invasion, and migration of human LSCC cells *via* the miR-218-5p/BMI1 axis.

The declining survival rates of patients with LSCC have been evident over the past years due to poor treatment efficacy (Marur & Forastiere, 2016). Numerous studies have identified the involvement of multiple lncRNAs in LSCC by several mechanisms (Wang et al., 2020; Xu & Xi, 2019; Yang et al., 2019). The current study sought to investigate the ceRNA mechanism of lncRNA CCAT1 in LSCC cells by regulating BMI1 *via* miR-218-5p (Fig. 6).

An elevated CCAT1 expression has been evident in the bladder cancer tissues (Zhang et al., 2019). For an analysis of the expression pattern of CCAT1 in LSCC, we detected the CCAT1 expression in Hep-2 and TU177 cells and HLCs and compared the results. Our findings presented a higher expression of CCAT1 in the Hep-2 and TU177 cells relative to HLCs. Next, we determined the effect of CCAT1 on LSCC cells by silencing CCAT1 in the Hep-2 and TU177 cells. Our results elicited that the proliferative ability of Hep-2 and TU177 cells was significantly weakened, while the migrative and invasive abilities were radically impaired after silencing CCAT1. Additionally, a previous study demonstrated the capacity of CCAT1 to facilitate LSCC cell proliferation and invasion (Zhuang et al., 2016), and our results were consistent with these findings.

Subsequently, we investigated the mechanism of CCAT1 in promoting LSCC cell proliferation, migration, and invasion. Initially, the subcellular localization of CCAT1 was predicted and validated in the cytoplasm. The binding sites of CCAT1 and its target genes were predicted using the Starbase database, and the result exhibited the targeted relationships between CCAT1 and miR-218-5p, which was further verified by the dual-luciferase assay. miR-218 is downregulated in LSCC (Zhang & Hu, 2017). Our results presented a reduced expression of miR-218-5p in Hep-2 and TU177 cells relative to the HLCs. In our study, an elevated expression of miR-218-5p was observed in the Hep-2 and TU177 cells after silencing CCAT1. LncRNA CCAT1 negatively regulates miR-218-5p in human retinoblastoma (Zhang et al., 2017). Similarly, our findings elicited a direct interaction of CCAT1 with miR-218-5p in Hep-2 and TU177 cells. The next step was to determine the function of miR-218-5p in the ceRNA mechanism of CCAT1 in LSCC. Firstly, a rescue experiment was conducted to introduce the miR-218-5p inhibitor in Hep-2 and TU177 cells with silencing CCAT1. The downregulation of miR-218-5p was subsequently identified, along with enhanced proliferation, invasion, and migration of Hep-2 and TU177 cells. Previously, overexpression of miR-218-5p could radically inhibit the proliferation, invasion, and migration of hepatocellular carcinoma cells (Yu et al., 2019). These findings together suggested that the downregulation of miR-218-5p could partially neutralize the effect of CCAT1 silencing on the proliferation, migration, and invasion of Hep-2 and TU177 cells.

We next sought to explore the downstream target gene of miR-218-5p. The overexpression of BMI1 has been evident in head-neck squamous cell carcinoma (Wang et al., 2017). Our results showed increased expression of BMI1 in Hep-2 and TU177 cells compared to that in HLCs. After predicting the presence of several binding sites of miR-218-5p and BMI1 on the Targetscan database, we further verified their targeted relationship by the dual-luciferase assay. Essentially, silencing CCAT1 could downregulate BMI1 in gastric cancer (Li et al., 2018a), whereas miR-218 suppresses the BMI1 expression in hepatocellular carcinoma (Wu et al., 2018). Our results demonstrated that a combination of silencing CCAT1 and miR-218-5p overexpression could terminally weaken the BMI1 expression in Hep-2 and TU177 cells while the downregulation of miR-218-5p annulled the reducing trend of BMI1 expression induced by silencing CCAT1. Conjointly, our findings depicted that miR-218-5p targeted BMI1 in Hep-2 and TU177 cells. To determine the role of BMI1 based on the CCAT1-mediated ceRNA mechanism in LSCC, an elevated BMI1 expression was evident in the Hep-2 and TU177 cells *via* transfection with pcDNA3.1 BMI1. The knockdown of BMI1 could critically inhibit the growth of hepatocellular carcinoma cells (Wu et al., 2019). Our findings revealed the enhanced proliferative, invasive, and migrative abilities in Hep-2 and TU177 cells after BMI1 overexpression, and herein revealed that the upregulation of BMI1, to some extent, could produce a conflicting effect to silencing CCAT1 on Hep-2 and TU177 cell proliferation, invasion, and migration. Additionally, CCAT1 functions as a definitive sponge for miR-218 in non-small cell lung cancer (Jin et al., 2020). An existing study documented the capacity of miR-218 to weaken the proliferative and metastatic abilities of esophageal squamous cell carcinoma cells *via* targeting BMI1 (Wang et al., 2015). Conjointly, our findings depicted that lncRNA CCAT1 promoted the growth of Hep-2 and TU177 cells by sponging miR-218-5p and thus regulating the BMI1 expression.

To conclude, our study identified a new ceRNA pathway in LSCC cells where lncRNA CCAT1 participated in the proliferation, migration, and invasion of LSCC cells by regulating miR-218-5p/BMI1. However, there is a limitation to our study is that we solely investigated the involvement of CCAT1 in the biological process of LSCC cells *via* miR-218-5p/BMI and whether CCAT1 and miR-218-5p could function as biomarkers in the early screening of human LSCC and exert prognostic effects on human LSCC remains elusive. Future studies are warranted for a comprehensive analysis of the mechanism of CCAT1 in human LSCC from the perspective of epigenetics.

## Additional Information and Declarations

### Data Availability

The following information was supplied regarding data availability: The raw data are available in the Supplemental File.
